# The association of marital relationship and perceived social support with mental health of women in Pakistan

**DOI:** 10.1186/1471-2458-13-1150

**Published:** 2013-12-09

**Authors:** Farah Qadir, Amna Khalid, Sabahat Haqqani, Zill-e-Huma, Girmay Medhin

**Affiliations:** 1 Department of Behavioural Sciences, Fatima Jinnah Women University, The Mall, Rawalpindi 46000, Pakistan; 2 Aklilu Lemma Institute of Pathobology, Addis Ababa University, P.O. Box 1176, Addis Ababa, Ethiopia; 3 School of Health in Social Science, The University of Edinburgh Medical School, Teviot Place, Edinburgh EH8 9AG, UK

**Keywords:** Marriage, Mental health, Social support, Scale validation

## Abstract

**Background:**

Marital circumstances have been indicated to be a salient risk factor for disproportionately high prevalence of depression and anxiety among Pakistani women. Although social support is a known buffer of psychological distress, there is no clear evidence as to how different aspects of marital relations interact and associate with depression and anxiety in the lives of Pakistani married women and the role of social supports in the context of their marriage.

**Methods:**

Two hundred seventy seven married women were recruited from Rawalpindi district of Pakistan using a door knocking approach to psychometrically evaluate five scales for use in the Pakistani context. A confirmatory factor analysis approach was used to investigate the underlying factor structure of Couple satisfaction Index (CSI-4), Locke-Wallace Marital Adjustment Test (LWMAT), Relationship Dynamic Scale (RDS), Multidimensional Scale for Perceived Social Support (MSPSS) and the Hospital Anxiety and Depression Scale (HADS). The interplay of the constructs underlying the three aspects of marital relations, and the role of social support on the mental health of married Pakistani women were examined using the Structural Equation Model.

**Results:**

The factor structures of MSPSS, CSI-4, LWMAT, RDS and HADS were similar to the findings reported in the developed and developing countries. Perceived higher social support reduces the likelihood of depression and anxiety by enhancing positive relationship as reflected by a low score on the relationship dynamics scale which decreases CMD symptoms. Moreover, perceived higher social support is positively associated with marital adjustment directly and indirectly through relationship dynamics which is associated with the reduced risk of depression through the increased level of reported marital satisfaction. Nuclear family structure, low level of education and higher socio-economic status were significantly associated with increased risk of mental illness among married women.

**Conclusion:**

Findings of this study support the importance of considering elements of marital relationship: satisfaction, adjustment and negative interactions which can be prioritized to increase the efficiency of marital interventions. It also highlights the role of social support in the context of marital relationships among Pakistani women. Furthermore, the study presents the etiological models of depression and anxiety with reference to the above.

## Background

Depression and anxiety are common and disabling conditions [1]. Strong research evidence indicates that women experience depression and anxiety more than men [2].

There is paucity of research in the field of mental health in Pakistan. However, the few studies that have been conducted have repeatedly reported that women have disproportionately higher rates of depression and anxiety compared to other developing countries [3,4]. Relationship and adjustment problems with husband and in-laws have been associated with attempted suicide [5] as well as common mental disorder (CMD) [6]. One study examining marital dissatisfaction and its relation to mental health reiterated the high rates of CMD among married women and attributed it to the women's perceived dissatisfaction from their marriage [7].

A systematic review based on 20 studies [4] carried out in both rural and urban parts of Pakistan reported the average prevalence of depression and anxiety in the community to be 34%. The range for women was 29-66% and for men it was 10–33. Factors perceived by women to affect their mental health were absence of a confiding relationship, marital disputes, verbal abuse by in-laws, too many children and financial difficulties [4].

Marital relation is one of the most frequently studied phenomena in the field of family and relationships [8]. Continued importance is placed upon the quality of marital relationship due to its impact on individual and family wellbeing [9]. Over the years the quality of marital relationship has been investigated as “satisfaction”, “adjustment”, “adaptation” and/or “happiness” [10]. Sometimes these terms have been used interchangeably and sometimes as complimentary elements of marital relationship quality [11-13].

Marital satisfaction and marital adjustment have been used interchangeably in research [14]. Although, there is no universally accepted definition of these constructs, their association with mental health and wellbeing is well documented [15-19]. In the absence of universally agreed upon definition of these concepts researchers are often motivated to use these terms according to their own interpretation of the concept [20]. Therefore, operationalizing variables becomes difficult leading to ambiguity in definition and affecting the validity of interpretations [21]. Hence, distinct and specific definitions are needed for accurate measurement which would help to compare and examine these concepts cross culturally.

The present study therefore hinges on a combination of two theoretical approaches in an attempt to study three distinct constructs of marital relations and to examine their interplay within the Pakistani cultural context. The first distinguishes between marital satisfaction and adjustment as distinct components of marital relations using intrapersonal and interpersonal distinction [22,23]. In this approach marital satisfaction is identified as an internal subjective characteristic of a person [24] and marital adjustment is considered to have dyadic properties referring to the interactions between spouses [12].

The second approach identifies relationship dynamics specific to negative patterns of interactions such as arguments that contribute to the quality of marital relations [14]. Combining these two approaches helps to develop a framework which proposes that being satisfied and/or well-adjusted in a marriage does not mean absence of negative interactions [25] and that if these negative interactions reach a certain threshold they could adversely contribute to the interpersonal adjustment within marriage which may decrease marital satisfaction [26]. The resultant framework is expatiated below.

It has been argued that marital satisfaction and adjustment are related although they represent different constructs and they should be measured separately [25]. In general, satisfaction is a state of happiness over pain [27] and it is a global assessment of the quality of a person’s circumstances based on their own selected norms. Although these norms are determined by cultural influences, satisfaction is internally decided. Thus if marriage is satisfying for one person it may not be automatically satisfying for another [8]. Marital satisfaction was initially thought to be represented by adjustment to spouse, marriage and marital relationship [28]. However, later marital satisfaction was recognised as a person’s subjective experience of the relationship [24,29]. On the other hand, marital adjustment is “the integration of the couple in a union, in which the two personalities are not merely merged, or submerged but interact to complement each other for mutual satisfaction and the achievement of common objectives” [12]. Furthermore, evidence indicates that marital adjustment even when reported by one partner indicates the perceived adjustment of the couple within the institute of marriage whereas marital satisfaction is the individual's own personal contentment within the relationship [30]. In our study the respondent reported marital adjustment as mutual interaction of the spouses, and for marital satisfaction they reported their subjective experiences.

Marital satisfaction may essentially not be composed of the elements that are simply the opposite of those that lead to dissatisfaction [8]. In fact it has been contended that where agreement in couples on major issues is important for marital adjustment, minor differences and even trivial anger exchanges if processed appropriately may broaden their perspectives within the dyad of the relationship and increase satisfaction [8,26]. On the other hand, studies showed that negative interactions like withdrawal; undermine marital satisfaction by diminishing positive factors of the relationship (e.g., trust, and commitment) in a marriage [31]. It is evident that the inclusion of elements of marital satisfaction and adjustment does not automatically imply absence/presence of negative interaction in the marriage, emphasizing that they must be studied separately.

Thus, we propose that different instruments should be used while researching the satisfaction, adjustment and interaction patterns in marital relationship research.

When taking concepts of marital relations as the object of research, it is essential to contextualize them. Therefore, in this paper we approach marital relations from the perspective of the Pakistani Muslim population. Pakistan is predominantly a Muslim state where marriages are highly influenced by religion. Though the religious sanctions require marriage to be based on mutual consent of the husband and wife, parental approval of both parties is considered necessary in Pakistan for a marriage to be socially and culturally approved. Like other countries in the region and unlike Western countries Pakistanis are less likely to endorse the boundary of relationships between the couple and their parents [32]. It is therefore not surprising that marital issues are shared with family members in hope of support [33], giving them a pivotal position in contributing to marital satisfaction, adjustment and interaction patterns for couples. This emphasizes the significant role of social support among Pakistani married women's marital relationship. This is supported by both theory and research [18,34].

There is little empirical evidence of the current state of marital relationships in Pakistan. This is partly due to the lack of availability of psychometrically sound instruments validated in this population. Keeping in view the unique background of Pakistani Muslim marriages it is important to first investigate the factor structure of relevant instruments that are to be used to measure martial circumstances in this population before embarking upon any endeavour to explain the state of marital relations and its association with social support and mental health of Pakistani women.

Our study was carried out on married Pakistani women. The objectives of this study were: (1) to assess the factor structure of Multidimensional scale for perceived social support (MSPSS) [35] (Zimet et al., 1988), Couple satisfaction Index (CSI-4) [36], Locke-Wallace Marital Adjustment Test (LWMAT) [37], Relationship Dynamic Scale (RDS) [38] and Hospital anxiety and depression scale (HADS) [39] and (2) to apply the structure equation model (SEM) to examine the associations between martial circumstances, perceived social support socio-demographic risk factors and mental health of these married women.

It is hypothesized that the increased marital satisfaction and adjustment enhances mental health of women and high levels of negative interactions can reduce marital adjustment, satisfaction and elevate the risk of Common Mental Disorder (CMD).

It is also hypothesized that social support will protect against mental health problems by enhancing marital satisfaction and adjustment and by helping to reduce negative interactions in marital relation.

## Methods

### Study setting and study design

A cross-sectional survey was conducted in Rawalpindi district of Pakistan. The Islamic Republic of Pakistan is a developing South Asian country with females accounting for 49.2% of its total population [40] which also reflects the population distribution of the district of Rawalpindi (48.8% females) [41]. The current study participants were recruited from urban and rural areas of the Rawalpindi district.

### Ethical considerations

This study was approved by Ethics Committee of Fatima Jinnah Women University Rawalpindi. Informed verbal/written consent was taken from the study participants. To ensure confidentiality interviews were anonymized using numerical codes.

### Recruitment of study participants

Eligibility criteria for the study was being a married woman at the time of data collection, within the age range of 17 to 65 years and being a resident of the pre-specified sites of Rawalpindi (i.e. Kallari, Askari and Jhanda Chichi). Divorced/Separated women or those diagnosed with severe medical or psychiatric conditions were not eligible.

The sampling frame was obtained from official Governmental lists which helped identify potential eligible participants. These were used with discretion only for the purpose of carrying out this research. Local contacts were used to facilitate access to the catchment areas. Door knocking technique was used to approach households. In one of the Urban slum areas (Jhanda Chichi) the local contact helped with the snowball sampling technique to cope with the respondents’ reluctance. Using this process of recruiting study participants information was collected from 277 married females (i.e. 67 from Kallari area, 96 from Jhanda area and 114 from Askari area). Out of 106 households approached in Kallari, 15 doors were locked from outside and 20 houses refused to participate in the study. In the Askari area 235 families were approached and 60 households did not respond to our door knocking, two attempts were made 11 households refused to participate and 2 families did not have eligible women.

### Data collection

Two trained female research assistants carried out the interviews under the supervision of a senior researcher. On average an interview took 33 minutes to complete. The protocol consisted of a structured socio-demographic questionnaire, the Self-Reporting Questionnaire (SRQ-20) [42] and Hospital Anxiety and Depression Scale (HADS) [39] were used to assess mental health, and women’s perceived social support was examined through Multidimensional Scale of Perceived Social Support (MSPSS) [35]. The three scales used to measure different aspects of marital circumstances were; the Relationship Dynamic Scale (RDS) [38], Locke-Wallace Marital Adjustment Test (LWMAT) [37], and the Couple’s Satisfaction Index-4 (CSI-4) [36].

### Description of the scales

#### Social support measures

1) Multidimensional Scale of Perceived Social Support (MSPSS) [35] is a 12 item measure of perception of support from family, friends and significant others. The respondents rate each item on a 7-point scale ranging from very strongly disagree (1) to very strongly agree (7). Hence, the total score ranges from 12 to 84. Previous studies have reported it to have good validity and reliability estimates [43,44]. The present study used the Urdu version of MSPSS which has strong psychometric properties [45].

#### Marital relationship measures

2) Locke-Wallace Marital Adjustment Test (LWMAT) [37] is a widely used self-report measure of adjustment in marriage. It assesses negative pattern of interaction between couples such as negative escalation, invalidation, negative interpretation, winner/loser, withdrawal and alternative monitoring. The scale scores range from 2–158. A score of less than a100 reflects marital distress in marital adjustment [46,47]. A wide range of research evidence supports the psychometric properties of this measure [48,49]. This scale was translated in Urdu using translation back translation method and pilot tested on 6 married women before applying it in the current study.

3) Relationship Dynamic Scale (RDS) [38] is an eight item measure which was developed to predict if a relationship is vulnerable to marital problems. The scale has shown good reliability and excellent validity [50]. In the current study we used the Urdu version of RDS [51].

4) Couple’s Satisfaction Index-4 (CSI-4) is a four item measure of relationship satisfaction [36]. The possible responses on each item range from 0 (not at all) to 6 (absolutely and completely). CSI-4 has robust psychometric properties [36]. The current study used the Urdu translated version of CSI-4 [52].

#### Mental health measures

5) Hospital Anxiety and Depression Scale (HADS) is a fourteen item scale developed to determine levels of depression and anxiety which are scored separately. This scale is scored on a 4 point likert scale (0 = not at all to 3 = most of the time) [39], generating a maximum score of 21 for each subscale [53]. The HADS scores may be interpreted as follows 0–7 (Normal), 8–10 (Mild), 11–14 (Moderate) and 15–21 as Severe [53]. HADS is a well validated instrument [54]. According to a review the sensitivity and specificity for both anxiety and depression sub-scales of HADS is approximately 0.80 [55]. Though HADS was not initially developed for community screening for depression and anxiety, however recently it has been extensively used and proven suitable for use in the general population in the developed [56,57] and the developing countries [58]. The present study used the Urdu translation [59] which has been used in a number of studies to screen for depression and anxiety [60-62].

6) Self Reporting Questionnaire (SRQ-20) is a 20 item instrument to screen psychiatric disorders. Every item has a yes (1) and a no (0) response format with a total score of twenty. The acceptable cut-off score for caseness of CMD is 8 and above [42]. The instrument has sound reliability and validity [42]. Its specificity ranged from 72-85% and sensitivity from 73-83% [63]. A translated Urdu version of SRQ-20 [64] was used in the current study. This scale has been previously used extensively in Pakistan [65,66].

### Data management and analysis

After cleaning the data basic characteristics of the study participants were summarized using frequencies. All scales indicated good internal consistency. A structural equation model was developed to test the relationship between perceived social support, marital relations and mental health. Before fitting full structural equation model, measurement models for each construct were investigated. Five measurement models were tested for the following unobserved variables (latent constructs): 1) two correlated latent variables, anxiety and depression, in which seven items of HADS were used as indicators of each construct 2) marital adjustment in which eleven items instead of original 15 items of Locke-Wallace Marital Adjustment test (LWMAT) were used as indicator variables (3) perceived social support (second level construct) for which three first level latent variables (friends, family, significant others) were used as indicator variables each of which (i.e. friends, family, significant others) were constructed using four indicator variables of MSPSS (4) relationship dynamics in which eight items of RDS were used as indicator variables and (5) couple satisfaction in which four items of CSI-4 were used as indicator variables. Each scale was tested for its fit to the data as it was hypothesized by its authors using confirmatory factor analysis approach. In case of poor fit, models were modified by excluding insignificant loadings of individual items and inclusion of correlated error terms. After deciding on the best fitting measurement model for each of the five scales, full structural equation model was fitted to evaluate the association of social support and martial circumstances with Depression and Anxiety of married women mediated through various alternative routes. In full structural equation model we tested for the following pathways (a) RDS with Anxiety and depression through SRQ-20, CSI-4 and LWMAT, (b) MSPSS with Anxiety and depression through RDS, LWMAT and CSI-4. Further it was tested if CMD (SRQ-20 score) associates with depression and anxiety. Moreover, the mediating pathway for increased CMD symptom between marital interaction and mental disorders (anxiety and depression) was assessed. Lastly, the effects of age, husbands education, family system, age at marriage, number of children and asset based socio-economic index (including ownership of TVs, VCD/DVD, computers, ACs, cars, house, servants in the house, and number of bedrooms, bathrooms, and foreign visits by the respondent) were investigated. In the process of developing measurement models and full structural equation model non-significant pathways and variables were removed from the models and the overall model fit indices were examined. Pathway associations for the prediction of psychological morbidity are expressed as crude and standardized regression weights. Correlations are reported for associations between unobserved variables. Overall model fit was assessed using the Tucker-Lewis Index (TLI), Root Mean Square Error of Approximation (RMSEA) and Comparative Fit Index (CFI). The Tucker-Lewis Index (TLI) [67] indicates the proportion of co-variation among indicators explained by the model relative to a null model of independence, and is independent of sample size. Values near 1.0 indicate good fit; those greater than 0.90 are considered satisfactory [68]. Comparative fit index compares the samples covariance matrix with the null model and its value ranges between 0.0 and 1.0 with values closer to 1.0 indicating good fit [69]. The Root Mean Square Error of Approximation (RMSEA) assesses badness of fit per degree of freedom in the model and is zero if the model fits perfectly; RMSEA values of less than 0.05 indicate close fit and 0.05 to 0.08 reasonable fit of a model [70].

## Results

### Background characteristics of study participants

Data was collected from a total of 277 married females within the age range of 17 to 65 years (M = 36.7 years, SD = 9.96 years). Background characteristics of these respondents are summarized in Tables 1 and 2. A large proportion of the study participants were above the age of 40 years, majority were house wives, age at marriage ranged from 13 to 43 years (M = 21.8 years, and SD = 4.32 years), 39% had more than 10 years of education, and 11% were employed outside home. Nuclear living arrangements were more frequent and family income ranged from Rs. 4000 to Rs. 250000 (M = Rs. 46984.77, SD = Rs. 47223.73). Majority of the respondents were married through family arrangements, 47% were married within the family and 22% had a history of abortion. Examination of asset based socioeconomic status showed that majority of them had moderate standard of living; 81.9% had TV, 63.9% did not have DVD, 39.7% had computer, 52.7% did not have ACs, 44.8% did not have their personal car, 66.1% could not afford to have servants in their house, 84.5% never had a foreign visit, 59.9% lived in their own home and 50.2% of the participants had a house with three to four bedrooms and attached bathrooms.

**Table 1:** Socio-demographic characteristics of study participants (N = 277)

**Characteristics**	**Response categories**	**Number *(%)***
**Age in years**	Less than 26 years	46(16.6)
	26–30 years	57(20.6)
	31–35 years	35(12.6)
	36–40 years	46(16.6)
	41 and years	93(33.6)
**Years of education**	No formal education	16(5.8)
	Grade 1–10	154(55.6)
	Above grade 10	107(38.6)
**Occupation of respondent**	Housewife	244(88.1)
	Working women	30(10.8)
	Students	2(.7)
**Husband’s education (n = 272)**	No formal education	13(4.7)
	Grade 1 to 10	244(88.1)
	Above grade 10	13(4.7)
**Husband’s monthly income PKR(n = 212)**	< 10001	55(19.9)
	10001–30000	107(38.6)
	30001>	50(18.1)
**Total earning members in family**	0-1 family members	173(62.5)
	2 & above	100(36.1)
**Total monthly income (n = 197)**	< 10001	38(13.7)
	10001–30000	72(26)
	300001>	87(31.4)
**Family system**	Joint family	113(40.8)
	Nuclear family	164(59.2)
**Presence of any physical or mental illness**	No	212(76.5)
	Yes	62(22.4)

PKR = Pakistani Rupee.

**Table 2:** Marital circumstances, (N = 277)

**Characteristics related to martial circumstances**	**Response categories**	**Number *(%)***
**Age at marriage**	Below 16 years	16(5.8)
	16–20 years	98(35.4)
	21–25 years	115(41.5)
	26 years >	46(16.6)
**Duration of marriage**	Less than 6 years	62(22.4)
	6–10 years	38(13.7)
	11–15 years	45(16.2)
	16–20 years	45(16.2)
	21–25 years	35(12.6)
	Above to 25 years	52(18.8)
**Decision of marriage**	Love based	22(7.9)
	Arranged by family	252(91)
**Type of marriage (n = 182)**	Not within family	52(18.8)
	Within family	130(46.9)
**Number of children ever born**	No children	28(10.1)
	1–2 children	92(3.2)
	More than 2	156(56.3)
**Most recent childbirth (n = 238)**	Normal	178(64.3)
	Caesarean	59(21.3)
**History of abortion**	No	156(56.3)
	Yes	61(22)

### Psychometric properties and factor structure of the scales

#### Multidimensional Scale for Perceived Social Support (MSPSS)

**Figure 1:**
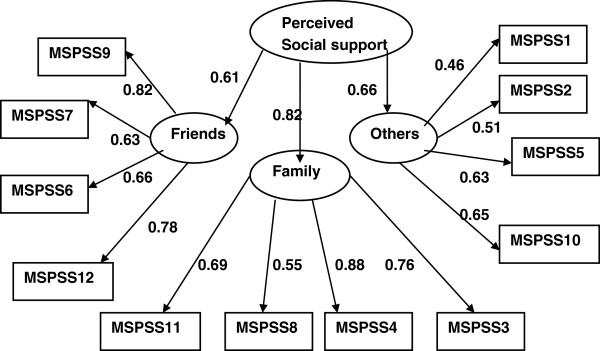
Factor structure of multidimensional scale of perceived social support (MSPSS) (The numbers attached to each variable name within each rectangular box indicates item number in the MSPSS scale, circle represents underlying factor for the set of indictor variables attached to it by an arrow, numbers on the side of each arrow represents standardized regression weights or standardized factor loading).

The factor structure presented in Figure 1 fits well to the data (Chi-square = 121.3 with 51 degrees of freedom and p-values < 0.001; TLI = 0.889, CFI = 0.927; RMSEA = 0.071(90% CI: 0.055, 0.088) and it is in line with the original structure of the scale. Indicators of perceived support from friends and significant others are less strongly correlated with the underlying construct compared to perceived support from family.

#### Locke Wallace Martial Adjustment Test (LWMAT)

**Figure 2:**
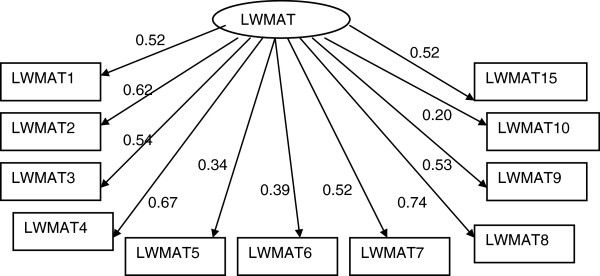
Factor structure of Locke-Wallace Marital Adjustment Test (LWMAT) (The numbers attached to each variable name within each rectangular box indicates item number in the LWMAT, circle represents underlying factor for the set of indictor variables attached to it by an arrow, numbers on the side of each arrow represents standardized regression weights or standardized factor loading).

The model summarized in Figure 2 fits relatively well to the data (Chi-square = 103.3 with 41 degrees of freedom and p-value < 0.001; TLI = 0.847, CFI = 0.898; RMSEA = 0.071(90% CI: 0.053, 0.088). The scale has uni-dimensional structure as suggested by the original authors of the scale. However, inclusion of items 11, 12 and 13 significantly affects the overall fit of the model and each of these items was not significantly correlated with the underlying construct. However, after removing these items the factor structure was stable and the remaining items had significant correlation with the construct although the magnitude of correlation between some of the items of the scale (item 10, 5 and 6) and the construct is small.

#### Couples Satisfaction Index (CIS-4)

**Figure 3:**
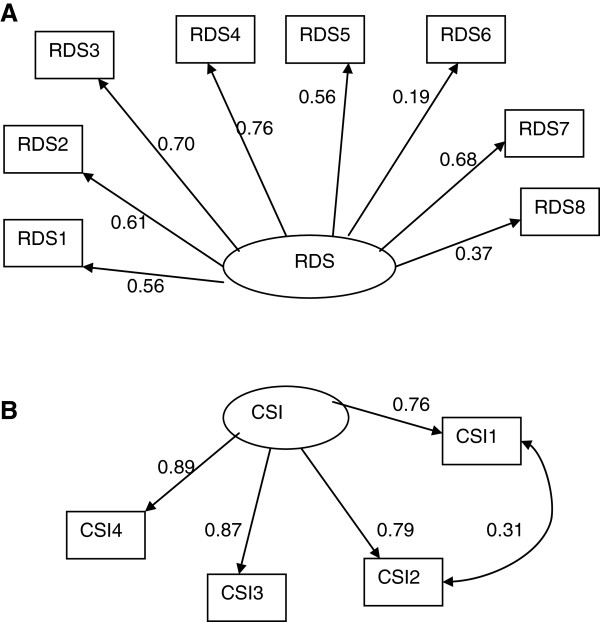
Factor structure of (A) Relationship Dynamics Scale (RDS) and (B) Couples Satisfaction Index (CSI-4) (the numbers attached to each variable name within each rectangular box indicates item number in their respective scale, circle represents underlying factor for the set of indictor variables attached to it by an arrow, numbers on the side of each arrow represents standardized regression weights or standardized factor loading).

The factor structure hypothesized for Couples Satisfaction Index (CIS-4) (Figure 3b) gave excellent fit to the data with Chi-square value of 0.13 with 1 degree of freedom and p-value =0.721; TLI = 1.00, CFI = 1.00; RMSEA = 0.000(90% CI: 0.000, 0.115). The uniqueness factors of item 1 and item 2 are significantly correlated indicating that these two items have some degree of commonality beyond what they share with the other two items of the scale.

#### Relationship Dynamics Scale (RDS)

The factor structure hypothesized for Relationship Dynamics Scale (RDS) (Figure 3A) gave excellent fit to the data with Chi-square value of 25.8 with 20 degrees of freedom and p-value =0.171; TLI = 0.977, CFI = 0.987; RMSEA = 0.033(90% CI: 0.000, 0.065). Item 6 is less important followed by item 8. However, a large proportion of variance within each of the remaining items was explained by the underlying Relationship Dynamics. Similarly, all the items used as indicators of Couples Satisfaction Index are highly correlated with the underlying satisfaction construct.

#### Hospital Anxiety and depression scale (HADS)

**Figure 4:**
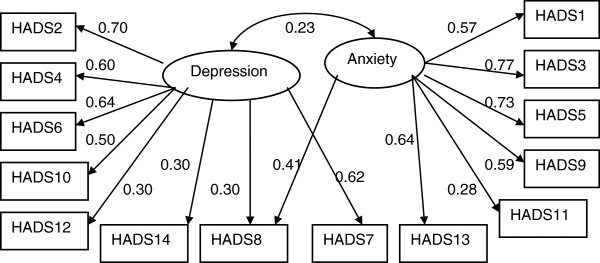
Factor structure of Hospital Anxiety and Depression scale (HADS) (the numbers attached to each variable name within each rectangular box indicates item number in the HADS, circle represents underlying factor for the set of indictor variables attached to it by an arrow, numbers on the side of each arrow represents standardized regression weights or standardized factor loading).

The model described in Figure 4 fits relatively well to the data (Chi-square = 199.3 with 75 degrees of freedom and p-value < 0.001; TLI = 0.815, CFI = 0.868; RMSEA = 0.078(90% CI: 0.065, 0.091). The scale has two correlated dimensions as it was suggested by the original developers of the scale. However, unlike the original suggestion item 8 loads significantly on both dimensions and item 7 loads on Depression but not on the Anxiety factor. There were also significant correlations between two pairs of items (i.e. uniqueness of item one with that of item 2 and uniqueness of item 11 with that of item 12). Assessing by the magnitude of standardized factor loadings items 8, 12 and 14 were less important indicators of Depression factor and item 11 was a less important indicator of the Anxiety factor.

#### Results from full structural equitation modelling

**Figure 5:**
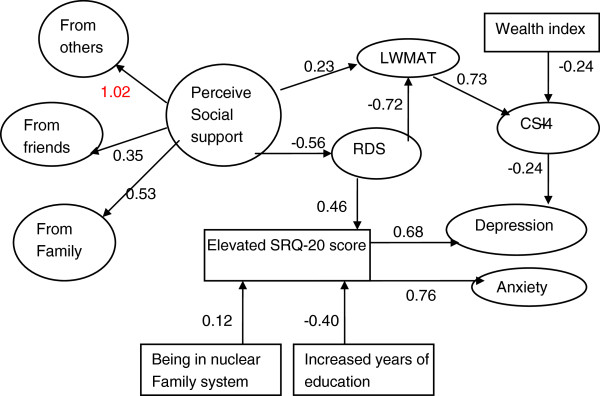
Structural equation model evaluating interrelationship of perceived social support, martial satisfaction, socio-economic situation and mental health status of married women (RDS = Relationship Dynamics factor; CSI = Couples Satisfaction index; LWMAT = Locke-Wallace Martial Adjustment; the numbers on the sides of the arrows are standardized regression coefficients or standardize factor loadings; variables within the rectangles are measured variables and variables within circle/oval are underlying constructs or factors expressed by measured indicator variables).

Full structural equation model (Figure 5) shows the associations of the socio-demographic characteristics, perceived social support, various aspects of marital relationship circumstances and the effects of these relationships on mental health status of married women. The overall model fits to the data reasonably well (TLI = 0.84, CFI = 0.85, RMSEA = 0.046 (90% CI: 0.042, 0.050)). Residing in nuclear family system and having less education are significantly associated with an increased risk of elevated CMD symptoms which in turn leads to an increased likelihood of Depression and Anxiety. Higher socioeconomic status does not have a significant direct effect on the levels of CMD symptoms or the likelihood of having Depression or Anxiety. However, it is a risk factor for Depression through its negative effect on the association with the level of the respondent's marital satisfaction. Furthermore, perceived higher social support reduces the likelihood of Depression and Anxiety by enhancing positive relationship as reflected by a low score on the relationship dynamics scale which decreases CMD symptoms. Moreover, perceived higher social support is positively associated with marital adjustment directly and indirectly through relationship dynamics which is associated with the reduced risk of depression through the increased level of reported marital satisfaction.

## Discussion

To the best of our knowledge the construct of marital satisfaction (CSI-4), adjustment (LWMAT) and negative interaction in marital relations (RDS), perceived social support (MSPSS) and their relationship with mental health of Pakistani married women (HADS) has not been studied previously. Therefore the present study sought to examine the interplay and associations between them using Structural Equation Modelling.

The results indicated that the three scales used to examine marital relations did in fact measure three separate yet interrelated elements of marriage. As hypothesized they inversely correlated with psychiatric morbidity of married women in Pakistan as assessed by HADS and SRQ-20. Increased marital satisfaction was protective against depression whereas social support had a buffering as well as a main effect on marital relations which in turn influenced the mental health of married women.

Residing in nuclear family system and having lower educational level are significantly associated with an increased risk of having elevated CMD symptoms which in turn leads to an increased likelihood of Depression and Anxiety.

### Social support as measured by MSPSS

The Urdu version of MSPSS in the present study replicated a three factor structure as proposed in the original study [35]. Similar structure was reported in a previous study conducted in Pakistan [71] and other Asian countries [72,73]. However, studies have reported one [45] or two [73] factor solutions as well.

Pakistani society encourages strong familial culture which was reflected in the factor structure of the social support scale. This is consistent with research on Indian immigrants in Britain [33] where married persons turn more frequently to family for support. In the current study the weakest correlation was observed between the latent construct of perceived social support and significant others. This is consistent with a study comparing Pakistani and Nepalese respondents living in Hong Kong. According to this study the Nepalese version of MSPSS demonstrated a three factor structure while the Urdu version of MSPSS confirmed a two factor structure; in which items pertaining to 'significant others' subscale were absorbed in the ‘family’ subscale [74]. Similarly another study among antenatal Pakistani women [45] showed a single factor structure for MSPSS. These studies collectively support the strong familial support system, however, support from friends and significant others has shown inconsistent results. These differences in factor structure can be explained in terms of difference in characteristics of the target population. Further exploration is suggested to establish the construct of perceived social support from a collective point of view.

### Marital adjustment as measured by LWMAT

The current finding supports that LWMAT is a unidimentional measure of marital adjustment as proposed previously [75]. Except for item 11 (Do you and your mate engage in outside interests together?), item 12 (What do you/does your mate prefer in leisure time?) and item 13 (Do you ever wish you had not married?) all other items contributed significantly to measuring the construct of marital adjustment. One previous study [76] excluded item 12 from the scale on the basis that “exploring leisure activities are more characteristic of couples who are friends”. Friendship and companionship may not be a desirable characteristic for marital adjustment in Pakistani culture where most of the marriages are arranged by families. A plausible explanation of item 11 not being a valid indicator of marital adjustment in the present study could be that in Pakistani cultural milieu women generally get little freedom to have leisure and social life outside home [77]. This reduces their chances of engaging in activities with their partners outside home. Items 13(Do you ever wish you had not married?) was perhaps a weak correlate because getting and staying married is religiously and culturally endorsed in Pakistani society for females. Also Pakistani women report to be committed to their marriage and respect the solemnity of the relation [7]. Previous research among Pakistani and Bangladeshi women living in UK [77] found that almost all unmarried women in their study explicitly stated that they will get married and already married ones had always expected it. It is perhaps impossible to envisage a life without marriage particularly for women who are informed by the parents and the society almost from birth that they belong to someone else and are to settle in another family. This could be one of the reasons that item 13 was not very relevant to Pakistani society.

### Marital satisfaction as measured by CSI-4

One dimensional factor structure of CSI-4 in the current study is in agreement with the findings of a previous study [36]. The significant co-variation between uniqueness of two items of CSI-4 (Degree of Happiness in the Relationship and Do you have a warm and comfortable relationship with your partner?) might be a function of the fact that unlike Western societies in Asian countries happiness is more a product of warmth and comfort in social relationships from which the individual derives pleasure where the self is perceived as part of the whole relationship [78].

### Dynamics of relationship as measured by RDS

The current study supports the original one factor structure for RDS as previously proposed [38]. Weak loading of item 6 of RDS (i.e. I think seriously about what it would be like to date or marry someone else) on its underlying construct might be explained as both ideas suggested as options are not culturally viable particularly for women in Pakistan. To date and to think about men other than the husband is considered blasphemous therefore to elicit a response to the exploration of alternatives the question should be rephrased to suit the Pakistani Muslim society.

### Depression and anxiety as measured by HADS

In the current study, a two factor structure emerged for HADS as proposed by the authors of the scale [39]. The two sub-scales were significantly correlated, this correlation could be explained by the known comorbidity between depression and anxiety [55,79,80]. Psychometric issues in HADS have been discussed in literature over time and several factor structures have been proposed [55]. The item analysis showed anomalous loading of two items of HADS (items 7 and 8) on depression and anxiety which is consistent with other previous studies [81]. Item 7 in the present study loaded on depression sub-scale but not on anxiety sub-scale. Similar results were reported previously [82] where item 7 (I can sit at ease and feel relaxed) loaded on the anxiety sub-scale as well as on depression sub-scale. Whereas in another study [83], this item loaded more strongly on depression subscale as compared to anxiety. Item 8 (“I feel as if I am slowed down”) loaded significantly on both dimensions in agreement with previous studies [54].

### Marital satisfaction, adjustment and negative interactions as distinct components of marital relations

The model built in the current study using structural equation model has important contributions to offer to the theory and interventions by focusing on the specific relationship elements and processes affecting mental health of married women in Pakistan. In the past researchers have used marital adjustment and marital satisfaction interchangeably with their own interpretation of the concepts [14,20,28]. In our study moderate correlation between marital adjustment and satisfaction implies that these are different but related constructs that should be measured separately, which has been previously suggested both empirically and theoretically suggesting that one may be used to predict the other rather than substituting one for the other [36].

The absence of significant correlation between CSI-4 and RDS in the present study also confirms previous postulation that they are distinct constructs. This contributes to the growing evidence that the elements of negative interaction are perhaps not mere opposite of happiness or satisfaction in marriage [8,10]. Furthermore, the association of negative interactions and marital adjustment confirms the proposition that studying marital satisfaction and adjustment does not automatically imply the absence/presence of negative interaction in the marriage and that they must be studied separately. Hence, these constructs can exist parallel to each other. These findings are particularly useful for clinical interpretations where clinicians are interested in the contribution of disagreement and satisfaction in marital conflicts. Moreover, different instruments may be used in research and by practitioners to determine if a marital relationship is non-distressed or if the couple experiences marital satisfaction.

In the current study increased negative interaction patterns and decreased marital satisfaction both independently contribute to the development of mental health problems. Whereas marital adjustment associates with depression through its relationship with marital satisfaction. This is in accordance with marital discord model of depression [19], suggesting that problems in marriage elevates the risk for psychological morbidity which leads to depression whereas marital satisfaction reduces the chances of experiencing depression. In SEM the pathway indicating the woman’s experience of increased negative interactions adversely affects their mental wellbeing which is in agreement with recent findings [84].

### Role of social support

The SEM model suggests that social support plays a role in determining the perceived quality of Pakistani women’s marital relationship. In keeping with Cohen’s theory [85] of mechanisms of social support and other findings [86], the role of social support in the current study is twofold; firstly it buffers the impact of negative interactions (RDS) in marital relationship to indirectly increase marital adjustment (LWMAT) and it also enhances the adjustment in marriage directly.

In Pakistani cultural context where boundaries between the relationship with parents and spouse are blurred [32], family plays a greater role as an influential source that affects the quality of marital interactions. In line with Goodwin and Cramer [33] the current study established a greater role of family members in providing social support for marital relations. Surra [87] argues that family members serving as a source of social support influence an individual by communicating their opinions about an individual’s actions. This process can in turn enhance or diminish the quality of marital interactions [87]. The support provided by the family can also enhance the quality of marriage by validating the relationship through asserting their worth as a couple, accepting them socially and assuring that they can work through their problems as a pair [88] this perhaps is one of the reasons why arranged marriages survived in communal cultures. Although there is evidence indicating negative role of families in marriages for women in Pakistan, however the present study indicates that when the family plays a positive role in a woman's married life and it is perceived as such it is likely to reduce risk of mental health problems for married women. As mentioned earlier in the background section that the prevalence for psychiatric morbidity for women is disproportionately high for Pakistani women and married women are more at risk because of dissatisfaction from marriage and problems with in laws. Our study is a step forward in encouraging interventions at individual, couple and social level to enhance support from the indigenous source of family. Further research needs to be conducted to get a perspective on how the same applies to men in their marriage.

### Role of education, family system and socio-economic status

Among the three demographic factors under discussion in the current study educational status has protective effect against mental health problems of married women. In the present study less education of participants was associated with increased CMD symptoms leading to depression and anxiety. This correlation has been reported in various studies [89] including studies in Pakistan where young women having higher educational level are found to be at lower risk for CMDs [90]. Secondly, living in nuclear family system was found to be protective against mental health problems in the present sample. Pakistan is a collectivist country where social relationships play a greater role in an individuals’ life. In the current study where family played an important role in providing social support, living with the spouse and/or children, away from other family members may negatively affect mental health which may lead to depression and anxiety. Previous studies in Pakistan have reported both living in nuclear and joint family system as being risk factors for CMDs [4,90]. More evidence is required and encouraged in future research to clarify this ambiguity.

Women reporting higher socioeconomic status (SES) were more likely to score low on marital satisfaction which in turn increased their risk for depression. However, a previous study examining the direct effect of SES on depression in Pakistan [90] reported a negative correlation. Further research is recommended in order to better understand the role of SES in marital relations and mental health.

### Limitations

The present study has important implications for marital relations, perceived social support and mental health of Pakistani married women. However, the results should be interpreted in the light of a few limitations. The cross sectional study design does not allow causal inference therefore prospective research is recommended to establish the temporal link between the above mentioned factors. Our sample size does not allow representation of regional differences. Furthermore, in our study men are not represented which does not permit a gender comparison. It is an essential aspect that needs to be addressed in future research. Having said this, the study is an essential first step in shedding light on important aspects to be looked at for improving mental health and marital satisfaction of Pakistani women.

## Conclusion

In summary the current findings shed light on the marital relationship processes and their etiological role in models of depression and anxiety by identifying specific aspects of marriage that may be addressed collectively or independently to improve the mental health of married Pakistani women. Furthermore, social support may be utilized as a resource to enhance marital relations and potentially reduce marital distress as a risk to women's mental health.

This study contributes to the theoretical models and intervention efforts. It fits well with theories of marital relations, mental health and social support, by allowing to capture the specific role played by individual elements of marital relations. The findings will help in refining the prevention programs for marital discord by targeting individual relationship processes directly to enhance the efficacy of interventions.

## Competing interests

We have no competing interests to declare.

## Authors’ contributions

The degree of author’s overall contributions are in the order of FQ, GM, SH, AK and Z-e-H. This being the case all authors have contributed significantly to the designing, data collection, data analysis, and preparation of the manuscript. All authors read and approved the final manuscript.

## Pre-publication history

The pre-publication history for this paper can be accessed here:

http://www.biomedcentral.com/1471-2458/13/1150/prepub
